# Topological transport and atomic tunnelling–clustering dynamics for aged Cu-doped Bi_2_Te_3_ crystals

**DOI:** 10.1038/ncomms6022

**Published:** 2014-09-23

**Authors:** Taishi Chen, Qian Chen, Koen Schouteden, Wenkai Huang, Xuefeng Wang, Zhe Li, Feng Miao, Xinran Wang, Zhaoguo Li, Bo Zhao, Shaochun Li, Fengqi Song, Jinlan Wang, Baigeng Wang, Chris Van Haesendonck, Guanghou Wang

**Affiliations:** 1 National Laboratory of Solid State Microstructures, Collaborative Innovation Center of Advanced Microstructures, and Department of Physics, Nanjing University, Nanjing 210093, China; 2 Department of Physics, Southeast University, Nanjing 211189, China; 3 Solid State Physics and Magnetism Section, KU Leuven, Leuven BE-3001, Belgium; 4 School of Electronic Science and Engineering, Nanjing University, Nanjing 210093, China

## Abstract

Enhancing the transport contribution of surface states in topological insulators is vital if they are to be incorporated into practical devices. Such efforts have been limited by the defect behaviour of Bi_2_Te_3_ (Se_3_) topological materials, where the subtle bulk carrier from intrinsic defects is dominant over the surface electrons. Compensating such defect carriers is unexpectedly achieved in (Cu_0.1_Bi_0.9_)_2_Te_3.06_ crystals. Here we report the suppression of the bulk conductance of the material by four orders of magnitude by intense ageing. The weak antilocalization analysis, Shubnikov–de Haas oscillations and scanning tunnelling spectroscopy corroborate the transport of the topological surface states. Scanning tunnelling microscopy reveals that Cu atoms are initially inside the quintuple layers and migrate to the layer gaps to form Cu clusters during the ageing. In combination with first-principles calculations, an atomic tunnelling–clustering picture across a diffusion barrier of 0.57 eV is proposed.

## Abstract (web summary)

Enhancing the transport properties of surface states in topological insulators is vital if they are to be incorporated into practical devices. Towards this end, Chen *et al.* study the transport in aged (Cu_0.1_Bi_0.9_)_2_Te_3.06_, where the ageing process suppresses bulk conductance by up to four orders of magnitude.

## Graphical abstract

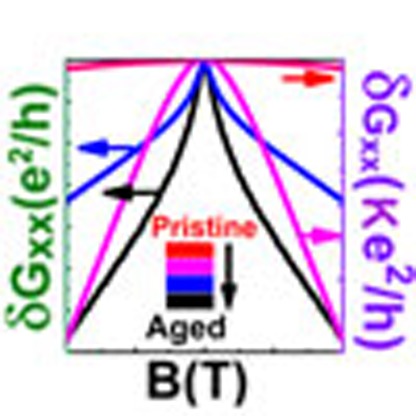

A substantial amount of attention has been paid to enhance the electronic transport through topological surface states (TSSs) in the study of three-dimensional topological insulators (TIs) of the Bi_2_Se_3_(Te_3_) families^1^,^2^,^3^,^4^,^5^. This is because TSS-mediated transport is commonly hindered by the conductance of bulk electrons due to imperfect electron-hole hybridization, intrinsic Se/Te vacancies or antisite defects in real TI samples^6^,^7^,^8^,^9^,^10^,^11^. Such bulk dominance often determines a non-TSS metallic transport in the TI devices, although some novel properties are expected, such as spin chirality, topological magnetoelectricity and a quantum Hall response.

Enormous efforts have been devoted to suppressing bulk conduction. For example, thickness reduction has been used as an effective strategy to enhance TSS transport^9^,^12^. However, even with an insulating mother crystal, the exfoliated ultrathin samples can still exhibit diffusive transport because the samples often suffer from undesired microfabrication contaminations^13^. In addition, samples always suffer from (local) roughness that inevitably leads to a non-uniformity in ultrathin TI layers prepared by molecular beam epitaxy^14^. Both aspects make the proper preparation of ultrathin TI layers laborious^5^. Another approach to suppress bulk conductance is to tune the TI samples (Fermi level) towards charge neutrality via the compensation of doped charges. Using ternary (Bi_2_Te_2_Se) or quaternary (Bi_2*x*_Sb_*x*_Te_3*y*_Se_*y*_) crystals has been successful because stoichiometric amounts of Bi_2_Te_3_ and Bi_2_Se_3_ contribute opposite types of carriers^7^,^15^,^16^,^17^. This has even led to some TSS-dominated TI crystals, for which two-dimensional (2D) Shubnikov–de Haas (SDH) oscillations have been observed^7^,^15^. However, an impurity band (IB) always appears to exist in such disordered crystals. Such impurity states may even couple the bulk and surface electrons in TI devices, resulting in a channel indicator of *α*=1/2 up to very large thicknesses^18^,^19^. Intentional doping with elements such as Ca, Sn and Tl has also been attempted^20^,^21^. Although doping by 0.1% can already suppress the bulk carrier concentration, a serious concern is that the Fermi level will further shift owing to the migration of the dopant atoms after the optimized TSS has been achieved^21^,^22^,^23^. Moreover, this sometimes leads to a topologically trivial crystal owing to a change in the spin orbit interaction^23^,^24^,^25^. Such subtle carrier compensation and IB-mediated coupling form critical obstacles to the current optimization of TI materials.

Ageing has been used to suppress the bulk transport of Bi_2_Se_3_ due to the absorption of oxygen^26^, which may stabilize the dopant atoms after a long period of time. In this work, we introduce an ageing method to (Cu_0.1_Bi_0.9_)_2_Te_3.06_ crystals whereby intense ageing leads to a great suppression of the bulk conductance of up to four orders of magnitude. The Fermi level is observed to move inside the bandgap, and no sizeable IB can be observed by the scanning tunnelling microscopy (STM). We successfully observe 2D weak antilocalization (WAL) and find a 3.3% increase in conduction from the TSS in the 40-μm-thick flake. Combined studies involving low-temperature transport, STM, transmission electron microscopy (TEM) and first-principles calculations reveal an atomic tunnelling–clustering picture for the Cu migration across a 0.57-eV-high diffusion barrier at the interface between the quintuple layers (QL).

## Results

### Suppressed bulk conductance after ageing

**Figure 1:**
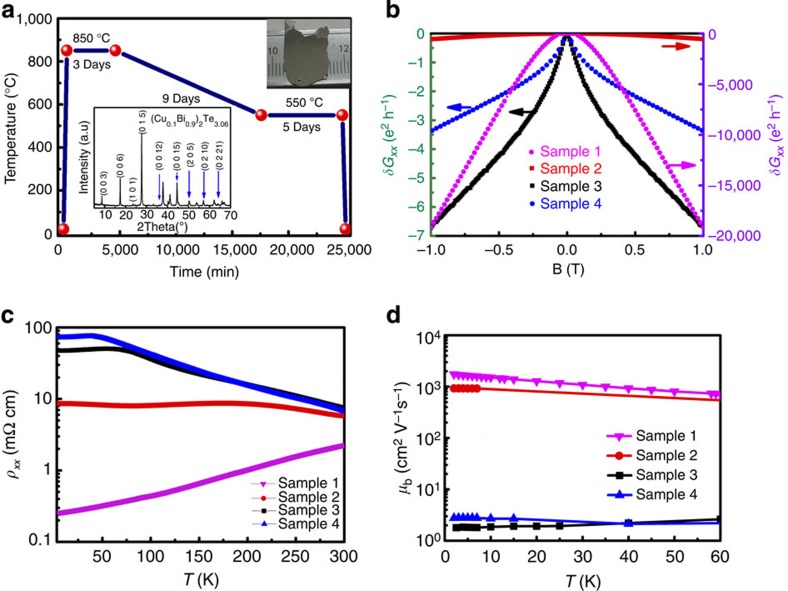
Preparation of (Cu_0.1_Bi_0.9_)_2_Te_3.06_ samples and their transport. (**a**) The temperature evolution during preparation of the crystals. An ageing process is then followed to prepare the low-mobility samples. Our samples are mechanically exfoliated; their typical size is shown in the inset at the top right. The bottom-left inset also shows the X-ray powder diffraction measurement, confirming the crystalline ordering of Bi_2_Te_3_. (**b**) The MC curves of the samples at 2 K. Sample 1 is the sample without ageing, Samples 3 and 4 are aged for 600 days, whereas Sample 2 is with a larger mobility and a smaller ageing period. The MC curves for Samples 1 and 2 are plotted on the right axis, and those for Samples 3 and 4 are plotted on the left axis. (**c**) The temperature-dependent resistivity of our samples. (**d**) The temperature-dependent mobility of the four samples. The intensely aged flakes, that is, Samples 3 and 4, have super low-mobility values. The sample parameters can be found in Table 1.

Cu-doped Bi_2_Te_3_ has been studied as a thermoelectric material for decades, where the intercalation and electrical inactiveness have been intensively discussed^23^,^27^,^28^,^29^,^30^. This material has recently been highlighted as a possible substitute for the topological superconductor Cu-doped Bi_2_Se_3_ (refs 23, 28, 31, 32, 33, 34), for which delicate carrier optimization and high-pressure studies have been carried out. We have attempted to search for superconductivity signatures in the samples but failed. Interestingly, we determined that the samples become band-insulating TI after being aged for over 1 year. All our crystals, which nominally have a (Cu_0.1_Bi_0.9_)_2_Te_3.06_ composition, are prepared using the melting method. The temperature evolution for the sample preparation process is presented in Fig. 1a. Four samples are considered with increasing ageing time and are referred to as Samples 1–4 (see the Methods section) in increasing order of ageing time. For comparison, a pristine Bi_2_Te_3_ crystal without any ageing is also prepared for comparison along similar routes. X-ray powder diffraction reveals a genuine crystalline structure with the no. 166 space group (inset of Fig. 1a, PDF Card 820358).

There are some transport indications of the TSS in the aged samples. The relative variation in the conductance of the samples as a function of an applied magnetic field between −1 and +1 T, that is, the magnetoconductance (MC), is plotted in Fig. 1b. A parabolic field dependence is found for Samples 1 and 2, while a tip-shaped MC feature near the zero fields is found for Samples 3 and 4. The parabolic MC curve is typical of normal metallic transport, which is influenced by the Lorentz force (bulk states). The MC curves for Samples 3 and 4 can be attributed to WAL. The WAL has recently been regarded as the signature (the TSS transport is pinned down in the next part) of TSS-induced transport^19^,^20^,^22^,^35^,^36^,^37^,^38^. Therefore, the above WAL dominance tends to indicate the enhanced TSS transport in aged samples, as also confirmed by the Onsager Phase obtained by SDH oscillations below.

We now attempt to check the bulk conductivity of the samples. Please see Fig. 1c for their temperature-dependent resistivities. The samples exhibit a metallic temperature-resistance dependence before ageing (Sample 1), where this dependence gradually becomes negative after ageing, as illustrated in Fig. 1c (Sample 4). In Sample 4, the low-temperature resistivity reaches a value of >100 mΩ cm. The curve reaches a plateau at low temperatures, which has been attributed to the diffusive TSS electrical transport^8^. Figure 1d also presents the temperature-dependent mobility of the samples, which decreases by four orders of magnitude after intense ageing (going from Samples 1–4). In addition, note that obvious SDH oscillations from the bulk electrons can be observed in Sample 1, while these sizeable SDH oscillations are greatly suppressed in the magnetoresistance (MR) curves of Samples 3 and 4. This confirms the pronounced suppression of the bulk electron mobility. Such suppression of the bulk conductance allows us to observe the TSS transport in bulk crystals.

### Evidence for enhanced TSS transport in aged samples

**Figure 2:**
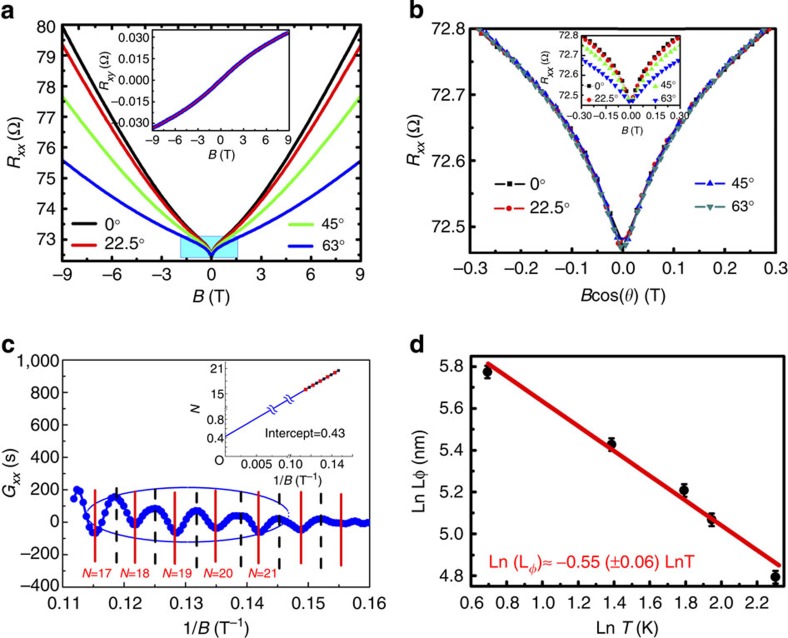
Evidence on the TSS transport in aged samples. (**a**) The angle-dependent MR curves of Sample 4. A characteristic dip of weak localization at zero fields can be observed. The inset is the Hall curve. (**b**) The MR curves plotted against the perpendicular component of the magnetic field. The inset shows the initial MR curves. The MR curves coincide after the field normalization. (**c**) The SDH oscillation and the Landau fan diagram. (**d**) The scaling of the dephasing lengths as a function of the temperatures. The ln-ln fitting gives a constant of 0.55±0.06, which reveals a typical 2D interference.

The angular dependence of the MC demonstrates the WAL character of the 2D TSS. Figure 2a presents the MR of Sample 4 measured along different directions at 2 K, where the tip shape of the WAL signal changes with increasing angle, as marked by the blue shadow and as illustrated in the inset of Fig. 2a. However, all low-field MR curves coincide after normalizing the magnetic field to correspond to its perpendicular component, as illustrated in Fig. 2b. This indicates that the observed WAL arises from a 2D electronic state^7^,^10^,^39^,^40^. The 2D WAL indicates the presence of a coherent surface state that is well confined within a depth that is much less than the dephasing length of our TI crystals with the suppressed mobility. A pindown of the TSS is obtained by extracting the SDH oscillations according to the methods^41^ shown in Fig. 2c. The Landau fan diagram in the inset gives an intercept of 0.43, nearly 1/2, which is typical evidence for Dirac transport. The phase is nearly 0 before ageing (Supplementary Fig. 1) This also excludes a possible trivial 2D electron gas. Considering a dephasing length of over 300 nm and a sample thickness of 40 μm, the electrical transport demonstrates the TSS-mediated electron transport in our TI crystals.

The 2D TSS transport can be further confirmed by a more detailed data analysis. We have fitted the WAL features at different temperatures according to the Hikami–Larkin–Nagaoka equation (Supplementary Fig. 2)^20^,^22^,^42^. The equation fittings provide the values of the dephasing lengths. The dephasing length decreases with increasing temperature, as shown in Fig. 2d. A ln-ln fitting is performed to identify the temperature scaling. Such fitting gives an exponential constant of 0.55±0.06 for the temperature dependence, which is typical of 2D electron interference^20^,^43^. All the above evidence points to an observable TSS-related electron transport in our aged Cu-doped Bi_2_Te_3_ bulk crystals.

**Figure 3:**
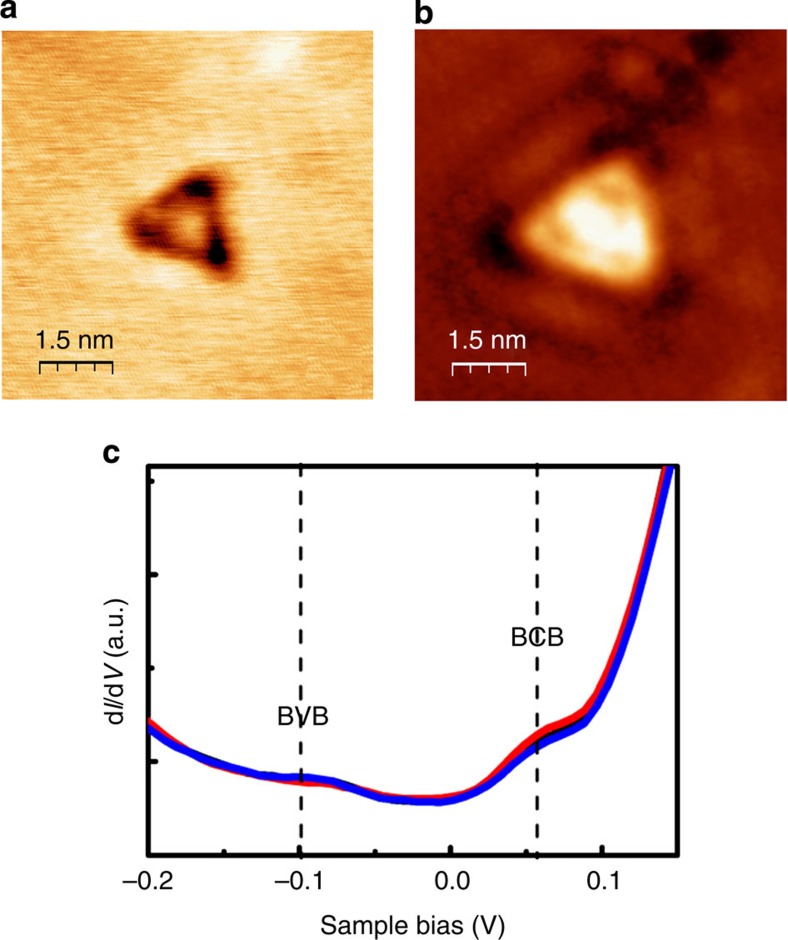
STM evidence on the TSS in Sample 4. (**a**,**b**)STM and spectrum images, respectively, around a defect, which demonstrate the electronic interference of a 2D surface state. (**c**) d*I*/d*V* curve for Sample 4. The black dashed line indicates the position of the Fermi level. Bulk valence band and bulk conduction band mark the positions of the valence and conduction bands, respectively, which are −0.10 and 0.06 eV higher than the Fermi level. The STM images are taken at a current of 0.5 nA.

STM also reveals the 2D electronic states. An STM topography image of a triangular-shaped defect in Sample 4 is presented in Fig. 3a. The corresponding map of the local density of states in Fig. 3b reveals the presence of complex wave patterns near the boundaries of the defect. Such patterns have long been interpreted as standing wave patterns that form owing to the interference of 2D surface state electrons, that is, 2D TSS electrons, which are scattered at the boundaries of surface defects. Figure 3c presents d*I*/d*V* curves recorded at different locations on the surface of Sample 4. The spectral features are in good agreement with previously reported spectra for Bi_2_Te_3_ (ref. 14). The Fermi level *E*_F_ is located within the bandgap at 0 meV, that is, 60 meV lower than the bottom of the bulk conduction band and 100 meV higher than the top of the bulk valence band. The width of the bandgap is in agreement with thermal activation-based calculations (Fig. 1c). This again confirms that our material is an insulator with a bulk bandgap and that the 2D electronic state is the TSS. We carefully collected the scanning tunnelling spectra at different positions for several times and obtained the typical d*I*/d*V* curves shown in Fig. 3c, where no obvious density of states can be found near the Fermi level or in the bandgap. This excludes the sizeable IBs, which is reasonable because Cu atoms are demonstrated to be nearly zero valence (Supplementary Fig. 3). All the above evidence reveals the fine protection and pronounced transport of the TSS even after the long period of ageing. Such enhanced TSSs are believed to be based on the band-insulating and IB-suppressed TI crystals.

The parallel conduction of the bulk state and the TSS should also lead to a bent Hall effect curve, as is observed in the inset of Fig. 2a^7^,^15^. The two-channel analysis of the Hall curve reveals that the ratio of the surface conductance to the overall conductance is 3.3%. The charge concentration of the TSS is determined to be 2.0 × 10^12^ cm^−2^, and its mobility is 1,400 cm^2^ V^−1^ s^−1^. The mobility of the bulk electron is only 2.4 cm^2^ V^−1^ s^−1^. This indicates that the TSS is preserved in spite of the suppressed bulk electronic states^6^. We also exfoliate the material and obtain some flakes with a thickness of ~100 nm, for which low-temperature transport is found to be totally dominated by the TSSs since its conductance is close to the surface conductance of the bulk sample. Because the materials have been aged for a long period of time, a few months of further exposure to air does not significantly influence the samples, in contrast to some other doped TI materials. Such optimized TI materials might be useful for the future fabrication of TI-based devices.

### Material analysis to reveal the dopant dynamics

As stated above, our Cu-doped Bi_2_Te_3_ crystals, which are highly metallic with a mobility of over 2,000 (sometimes 20,000) cm^2^ V^−1^ s^−1^ (see Sample 1), become electronically insulating with a very low mobility of 2.4 cm^2^ V^−1^ s^−1^ after intense ageing (Sample 4). Despite the high level of disorder, the TSS arising from the crystal symmetry survives, as demonstrated by the electron transport provided by the 2D electronic state. Then, a question arises concerning what occurs that subsequently leads to the mobility suppression of the bulk electronic states during the period of ageing. We first check the possible occurrence of oxidation by X-ray photoemission spectroscopy (XPS). The XPS data are collected while etching the sample with an Ar ion beam. The analysis of the XPS data reveals that all the O signals are confined to <20 nm from the surface. Oxidation cannot induce disorder in the bulk of the crystals with a thickness of 40 μm. Oxidation is therefore excluded as a possible cause of the observed mobility suppression. H_2_O absorption can also be excluded because it is again not possible to account for the suppression of the complete crystal. Such conjecture is reasonable because the crystals are sealed in vacuum during ageing.

**Figure 4:**
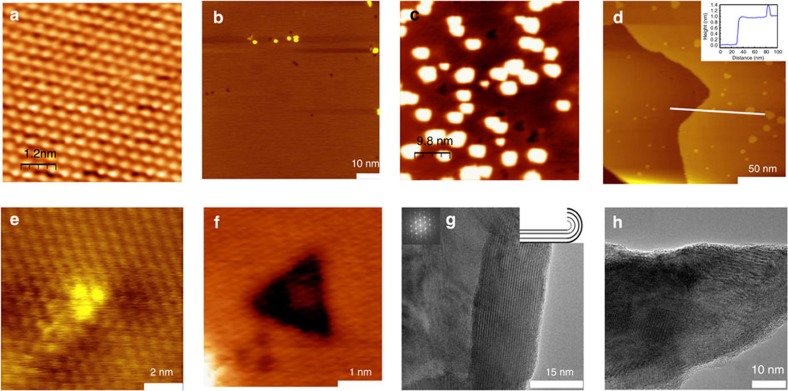
Defect characterization by STM and TEM. (**a**) STM image of a pristine Bi_2_Te_3_ crystal with a scale bar of 1.2 nm. (**b**,**c** STM results from two flakes extracted from the unaged and aged samples of (Cu_0.1_Bi_0.9_)_2_Te_3.06_, corresponding to (**b**) Sample 1 and (**c**) Sample 4, respectively. The STM images are taken at a current of 0.5 nA. Two types of defect features appear in the images (the light islands and the dark triangular defects). One may observe that the light islands are dominant in **c**. (**d**) Large-scale scanning of Sample 4, confirming the dominance of the light islands. A linear plot of Sample 4 is shown in the inset of **d**, indicating the white islands’ heights of ~0.4 nm, typical for Cu clusters. (**e**) Atomic-resolution image of the smallest light islands, which is obviously a 3-atom cluster, Cu_3_ clusters. (**f**) High-resolution image of the dark triangle, whose depth is 0.04 nm. (**g**) HRTEM image of the unaged samples, where the 1 nm-period fringes appear along the edge and the hexagonal lattices appear in the whole flake. Its model is illustrated by its inset. (**h**) Typical HRTEM image of Sample 4, where the crystals are much smaller.

The STM measurements are able to reveal the presence of defects and yield more information on the atomic dynamics of the Cu dopants during the doping and ageing processes. Figure 4a presents an atomically resolved STM topography image of an atomically flat terrace of a freshly cleaved (*ex situ*) pristine Bi_2_Te_3_ flake. It can be observed that the surface is free of defects and adsorbates. After doping with Cu atoms, two novel features are found at the sample surface (Fig. 4b–d): nanometre-sized islands (brightly coloured particles in Fig. 4e) and triangularly shaped defects (dark coloured particles in Fig. 4f). The number of Cu dopants is found to scale with the number of observed triangularly shaped defects. The islands are also observed on samples that are cleaved *in situ* (see STM topography images in Fig. 4b,d). Such islands are absent on the surface of pristine Bi_2_Te_3_. We therefore conclude that both the islands and the triangularly shaped defects are induced by the Cu doping. The triangular defects can be attributed to the Cu dopants inside the QLs,^44^ and the bright islands can be identified as Cu clusters.

**Figure 5:**
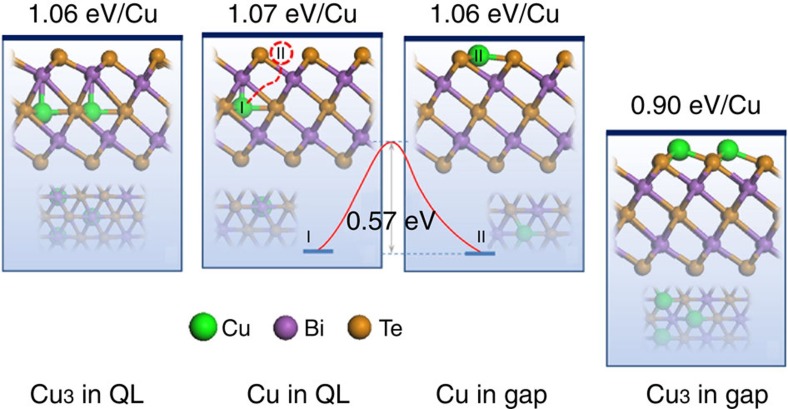
Modelling the ageing process. The atomic dynamics of Cu atoms from calculations. The four images illustrate the formation energies of Cu_3_ clusters inside QLs, individual Cu inside QLs (position I), individual Cu between QLs (in gap, position II) and Cu_3_ clusters between QLs (in gap). In each image, the top part is the side view, and the bottom greyer part is the top view. The formation energies are marked on the top. There is a transient state whereby the Cu atom is set on the QL interfaces that produces a 0.57-eV barrier separating the Cu atoms in the two states.

The STM topography image of a typical Cu_3_ cluster is presented in Fig. 4e. The height profile in Fig. 4d reveals the presence of a 1-nm-high terrace and a 0.4-nm-high island, which is a typical height for atom-sized Cu islands. Cu atoms can reside in three different positions in the crystals, that is, inside the QLs (position I in Fig. 5), in between the QLs (position II) and at the interfaces of the two positions. Cleavage always occurs between QLs; therefore, the observed islands can be assigned to Cu clusters that reside in between the QLs. Changes in the two features can then be interpreted in terms of the dynamics of the Cu atoms in the Bi_2_Te_3_ crystals. Note that Fig. 4b is obtained for a sample without ageing, while Fig. 4c,d are obtained for an aged sample (Sample 4). We can see that the bright islands resulting from the presence of Cu clusters become more abundant after the ageing process. The measurements thus indicate that the Cu atoms, which are initially inside the QLs after doping, migrate to the gaps in between the QLs during the ageing process. Subsequently, the Cu atoms then aggregate to form the Cu clusters that are observed in Fig. 4b,c.

The structural analysis by high-resolution TEM (HRTEM) demonstrates the degradation of the crystalline quality after ageing. The HRTEM does not visualize the Cu clusters owing to the difficulty in properly aligning the crystal. Figure 4g presents a typical HRTEM image of the pristine Bi_2_Te_3_ and of the (Cu_0.1_Bi_0.9_)_2_Te_3.06_ before ageing. We see that the crystal flakes can be very large, up to over 200 × 200 nm. Taking the HRTEM images along the edge of the flake, we are able to observe some parallel fringes with a lattice spacing of 1 nm. These are the (003) fringes that correspond to the growth direction of the Bi_2_Te_3_ QLs. Moreover, we can also observe the ideal hexagonal lattice in other regions of the flake in the HRTEM images. Here, we only present the electron diffraction pattern in the inset due to the limited resolution. The HRTEM analysis thus reveals the very good crystalline structure of our samples before ageing. The appearance of the (003) fringes along the edge is due to the natural curling of the thin flakes, an effect that also occurs for graphene flakes^45^,^46^. After ageing, we only observe much smaller crystalline domains, as illustrated in Fig. 4g. The typical dimensions of the domains are ~5 nm, but were found to be 10–20 nm in some cases. The HRTEM results provide evidence for the decay of the crystalline order during the ageing process. The observed poor crystalline order will naturally lead to strong electronic disorder and thus accounts for the observed pronounced suppression of the mobility of the bulk electrons.

### Interpreting the atomic dynamics by calculations

First-principles density functional theory (DFT) is used to describe the atomic dynamics of Cu dopants in Bi_2_Te_3_ (refs 47, 48). Geometrical optimization is performed for all possible positions of the Cu atoms, including inside the QLs (position I), between the QLs (position II) and at the interface (the ‘transient’ state). Initially, we check the positions inside the QLs, where Cu atoms occupy the substitutional positions (Bi or Te) and interstitial positions, respectively. The interstitial positions in the Te layer are found to be energetically favoured, as shown in Fig. 5. Next, a second calculation is carried out for the positions in between the QLs, which shows that the Cu atom prefers to adsorb on one side of the gap, in a hollow site of three Te atoms, as shown in Fig. 5. In addition, the formation energy here is very close to those of the atoms inside the QLs. Finally, the migration path of the Cu atom diffusion from the QLs to the gap is calculated. As illustrated in Fig. 5, a significant reaction barrier as high as 0.57 eV is located, which limits the direct exchange of Cu atoms in between them despite their similar binding energies.

Because the Cu atom is rather small compared to the QL gap^49^,^50^,^51^,^52^, the possibility of the formation of Cu clusters also needs to be considered. As indicated above, we also calculate the probability of Cu cluster formation. The result reveals that the formation of Cu_3_ clusters in between the QLs is preferred with a 0.16 eV per Cu atom smaller binding energy than for an individual dopant atom. In contrast, the system with Cu_3_ clusters exhibits a total energy similar to the energy of the system with separate Cu atoms inside the QLs. We should note that the dopant concentration may have some influence on the diffuse barrier. However, the proportion of Cu atoms in (Cu_0.1_Bi_0.9_)_2_Te_3.06_ in our experiment is rather small, indicating that the Cu atoms are very unlikely to diffuse in the forms of dimers or clusters; thus, this calculation model should still be applicable for a slightly denser dopant concentration, and the diffusion barrier will not change by much.

Based on above calculations, a clear ‘tunnelling–clustering’ picture with a diffusion barrier is revealed, where Cu atoms can migrate freely both in and between the QLs, while they have to overcome a 0.57-eV-high barrier when crossing the interface. During the long ageing period, the dopant atoms diffuse inside and between the QLs and frequently challenge the barriers. Because the formation of Cu clusters between the QLs determines the final direction of the atomic dynamics towards the QL gaps, Cu atoms in the QLs will gradually climb over the barrier and form clusters between the QLs, as observed in the experiment. The crystalline quality of the QL will degrade owing to the migration of a vast amount of Cu atoms during the ageing process, finally leading to smaller crystalline domains and to the strongly suppressed mobility of the bulk electrons. In contrast, the calculation also shows very small charge transfer, <−0.1 e per Cu atom both before and after ageing. Such small charge transfer compensates the subtle p-type carrier in the intrinsic Bi_2_Te_3_ crystal and leads to the formation of a band insulator. Because the Cu atom is smaller than Te and Bi atoms, we can expect that bigger atoms with the same outer atomic configuration, such as Ag or Au, will have higher diffusion barriers than does Cu, which also supports our choice of Cu dopant. The migration direction can also be confirmed by the charge concentration. The DFT calculations also provide the amount of charge transfer, which is −0.072 e per Cu atom in between the QLs and −0.043e per Cu atom inside the QLs. The pristine Bi_2_Te_3_ crystal is p-type doped, while the Cu-doped Bi_2_Te_3_ crystal before ageing is n-type doped and the aged Cu-doped Bi_2_Te_3_ crystal remains n-type doped as found in the experiments. Such small charge transfer may compensate the subtle intrinsic carriers in the Bi_2_Te_3_ crystals. A full story based on the atomic tunnelling–clustering is thus made.

Some previous work observed Cu atoms between the QLs, which is confirmed to be energetically favoured by our calculation. The present situation, whereby the Cu atoms are initially distributed both inside and between the QLs, can be interpreted by the melting condition during the crystal preparation. We suggest that annealing is not sufficient to achieve the most stable configuration. The metastable doping condition in the samples leads to the above ageing/diffusion process and to the final suppression of the bulk conductance both in the carrier concentration and mobility, that is, the insufficient annealing of the crystal accidentally causes the optimized TSS transport after ageing. We also note that ageing has been studied in TI samples^53^,^54^,^55^,^56^, and the suppressed bulk conductance has been observed^53^. Ultra high vacuum ageing leads to some topologically trivial 2D electron gas, and atmospheric ageing leads to some suppressed bulk conductance. We simultaneously observe the bulk suppression and TSS transport despite the fact that the present ageing process is performed in vacuum. As demonstrated by the DFT calculation above, the Cu migration within the crystal can account for both the bulk mobility suppression and the n-type-direction shift of the Fermi level.

In conclusion, the 2D electron transport provided by the TSS is observed in bulk crystals of aged (Bi_0.9_Cu_0.1_)Te_3.06_, as demonstrated by measurements of the WAL effect. The mobility of the bulk carriers is suppressed by four orders of magnitude during the ageing process. Both the STM and the electrical measurements support a Fermi level inside the bandgap. The ageing method therefore leads to an optimized band-insulating TI crystal and appeals to a free-of-IB crystal. STM visualizes the novel defect features of Cu dopants and their dynamics during the ageing process, based on which the details of the ageing process are further revealed by *ab initio* calculations. These calculations suggest that there exists a diffusion barrier at the interface of the Bi_2_Te_3_ QLs. During the ageing process, Cu atoms freely migrate inside the QLs and frequently hit the barrier. The dopant atoms will also form clusters in between the QLs, leaving disorder within the QLs. This leads to a pronounced mobility suppression of the bulk electrons, finally allowing the observation of the TSS-related electron transport in bulk crystal samples.

## Methods

### Sample preparation

All our crystals were prepared by the melting method, during which mixed high-purity Bi, Te and Cu powders (99.999% purity, Alfa Aesar, with a molar ratio of 2:3:0.15) were sealed in a silica ampoule. The material was then heated to 850 °C for 3 days while being slowly stirred at a speed of 5 turns per minute. After slowly cooling to 550 °C in 9 days, the material was annealed at 550 °C for 5 days (the melting curve is quite common in the present Cu-doped Bi_2_Se_3_(Te_3_) studies, but the temperature and annealing period may be further optimized if one hopes to remove the instability (ageing dynamics) described in this study). The obtained crystals were then cleaved with a razor blade, resulting in large crystalline flakes with a thickness ranging from 1 to 500 μm and lateral dimensions of a few millimetres. An ageing process was then performed after again sealing the flakes in vacuum. A typical ageing period was 600 days. After exposing the aged flakes to air, we could obtain the sample flakes, as described in Table 1. Initial Raman tests shown in Supplementary Fig. 4 also confirm Cu doping and ageing make no obvious influence on the crystalline order.

### Sample characterization

X-ray powder diffraction measurements were performed on a CAD4/PC (Enraf Nonius) diffractometer and on an X'Pert PRO (Philips) diffractometer, revealing an excellent crystalline order according to the no. 166 space group (inset of Fig. 1a, PDF Card 820358). An analysis with an inductively coupled plasma mass spectrometer was used to determine the chemical composition of the (Cu_0.1_Bi_0.9_)_2_Te_3.06_. For the electrical transport measurements, six probe electrodes were attached to the samples with room temperature cured silver paste. The transport properties were measured by a Quantum Design PPMS-16 system. STM and scanning tunnelling spectra experiments were performed using an Omicron Nanotechnology setup and a UNISOKU USM-1600 30 mK setup. The Omicron STM was operated at 4.5 K and at a base pressure of 10^−11^ mbar, where the samples were annealed in ultra high vacuum before measurement. In the UNISOKU STM, *in situ* cleavage was performed in ultra-high vacuum (at a base pressure of 1.0 × 10^−10^ mbar) at room temperature before the STM measurements at 4 K. The TEM images were obtained with an FEI TECNAI F20.

### Calculations

The spin-polarized DFT calculations are performed using the pseudopotential plane-wave method with projected augmented wave^57^ potentials and a Perdew–Burke–Ernzerhof-type generalized gradient approximation^58^ for exchange-correlation functionals, as implemented in the Vienna *ab initio* simulation package^59^. The plane-wave energy cutoff is set to be 400 eV. To investigate the behaviours of the dopant Cu at the concentration of the experimental conditions in bulk Bi_2_Te_3_, we use a 3 × 3 × 1 supercell containing 54 Bi and 81 Te atoms. Different possible positions of Cu atoms inside the QLs (position I) and between the QLs (position II) of Bi_2_Te_3_ are considered. A gamma-centered 3 × 3 × 3 k-point mesh within a Monkhorst-Pack scheme is adopted for integrations over the Brillouin zone^48^. All atomic positions are fully relaxed without any symmetry constraint until the Hellmann–Feynman force on each ion and the total energy change are <0.01 eV Å^−1^ and 1 × 10^−4^ eV, respectively. To evaluate the stability of different Cu dopant positions in Bi_2_Te_3_, we calculate the formation energy, which is defined as *E*_f_=(*E*[Bi_2_Te_3_Cu_*n*_]−*E*[Bi_2_Te_3_]−*nE*[Cu])/*n*, where *E*[Cu] is the energy of a Cu atom in the bulk phase, *E*[Bi_2_Te_3_] is the energy of the pure Bi_2_Te_3_ in a supercell and *E*[Bi_2_Te_3_Cu_n_] is the energy of the Cu-doped Bi_2_Te_3_. The climbing-image nudged elastic band method incorporated with spin-polarized DFT is used to find the minimum energy path and locate possible transition states^47^.

## Author contributions

F.S., B.W. and J.W. conceived of and coordinated the work. T.C. and Xu.W. prepared the samples and performed the transport measurements. Q.C. and J.W. performed the simulation. K.S., W.H., Z.L., S.L. and C.V.H. performed the STM measurements. Xu.W. and B.Z performed the electron microscopic measurements. Xi.W., F.M., Z.L. and G.W. participated in the discussions. F.S., K.S. and J.W. wrote the paper. All the authors commented on the manuscript.

## Additional information

**How to cite this article:** Chen, T. *et al.* Topological transport and atomic tunnelling–clustering dynamics for aged Cu-doped Bi_2_Te_3_ crystals. *Nat. Commun.* 5:5022 doi: 10.1038/ncomms6022 (2014).

## Supplementary Material

Supplementary Information Supplementary Figures 1-4.

## Figures and Tables

**Table 1:** Parameters of the (Cu_0.1_Bi_0.9_)2Te_3.06_ samples.

**Samples (name)**	**Size (mm × mm × μm)**	**Resistivity at 2 K (mΩ cm)**	**Mobility (cm**^**2**^** V**^**−1**^** s**^**−1**^**)**	**Resistivity at 300 K (mΩ cm)**
Sample 1	2.3 × 1.1 × 20	0.3	1,980	1.8
Sample 2	2.4 × 1.2 × 50	8.3	925.1	7.6
Sample 3	2.2 × 1.0 × 32	48.0	1.8	9.8
Sample 4	4.1 × 1.0 × 40	72.4	2.6	10.2

Sample 1 is free of ageing. Samples 3 and 4 are aged for 600 days. Sample 2 has half of the ageing extent of Samples 3 and 4.
