# Supplementary material for: Topological transport and atomic tunnelling–clustering dynamics for aged Cu-doped Bi2Te3 crystals
Source: Nat Commun. 2014 Sep 23;5:5022. doi: 10.1038/ncomms6022 (PMC4199107; doi:10.1038/ncomms6022)
Supplement: Supplementary Information — Supplementary Figures 1-4. [file ncomms6022-s1.pdf]

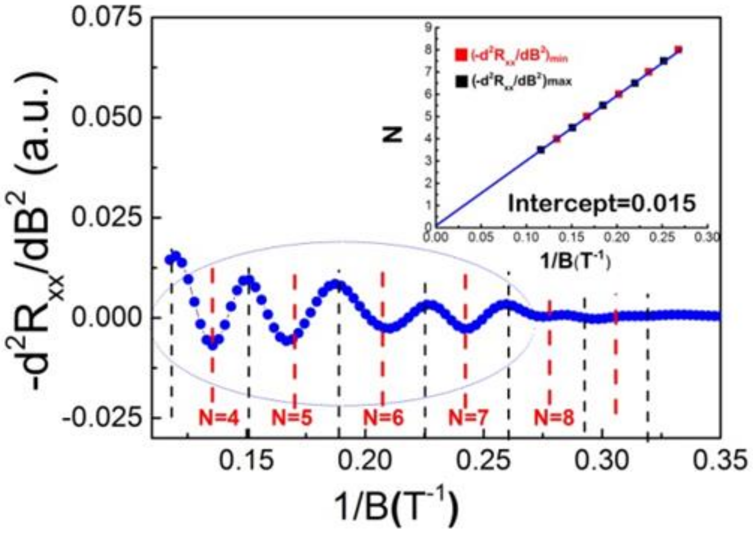

Supplemenatry Figure 1 – Comparison of the Shubnikov–de Haas oscillation of our samples before aging.

Here we show we can obtain the Shubnikov–de Haas (SDH) oscillation in the MR of sample 1. Please see Supplemenatry Figure 1 for the result. A periodic oscillation appears in its dependence on the inverse field, which is a typical SDH signature. Here we show the minus second derivative MR to assign the Landau index of the electronic state. We use the local minimum of the y axis to mark the integer index. This is reasonable because the conductance can be described as

$$G_{xx} = \frac{R_{xx}}{R_{xx}^2 + R_{xy}^2}$$

where G_{xx} is the conductance, R_{xx} and R_{xy} are the resistance and Hall resistance respectively. In our highly-metallic samples before aging, it is seen that $R_{xx} << R_{xy}$ .Therefore, the evolution of G_{xx} is simultaneous to that of R_{xx}, essentially to that of $-d^2R/dB^2$. Therefore, the assignment of the Landau index is reasonable. Please see the inset of Supplemenatry Fig.1, where the Landau Fan diagram is shown. The curve's intercept is 0.015, nearly zero, which prove this is a topologically-trivial state in the unaged samples.

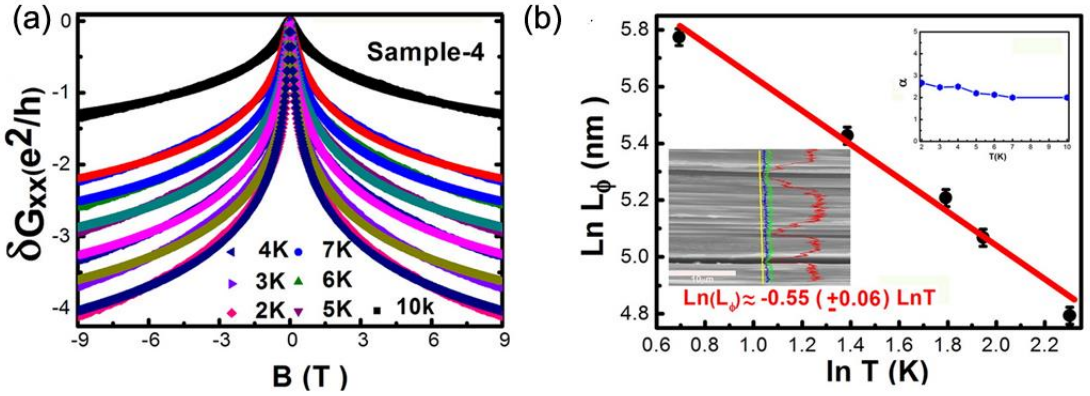

Supplementary Figure 2 – Temperature-dependent weak antilocalization measurement and analysis.

Supplemenatry Figure 2(a) shows the weak antilocalization (WAL) features at different temperatures, where all the curves are nicely fitted according to the Hikami-Larkin-Nagaoka equation. The equation fittings provide the values of dephasing lengths and channel numbers. The dephasing length decreases while the temperature increases as shown by Supplemenatry Fig. 2(b). A ln-ln fitting is carried out to identify the temperature scaling. Such fitting gives an exponential constant of 0.55±0.06 for the temperature dependence, which is typical of two-dimensional (2D) electron interference. The obtained channel number α is shown in the right inset of Supplemenatry Fig. 2(b), nearly 3 at low temperatures. It decays to 2.3 at higher temperatures due to the possible coupling. The value of around 3 indicates the presence of 6 surface state (SS) channels, i.e. 6 surfaces. This can be understood when considering the left inset of Supplementary Fig. 2(b), which is an SEM image of the sample’s sidewall. We observe the presence of some crack-like features on the side wall, which can also be observed for other layered crystals. Such features may be related to the mechanical cleavage and expose some more surfaces. Here we note that the 2D WAL only reflects there is a 2D electronic state on the surface, which is also possible to be trivial 2D electron gas. In such background, only after combining the SDH oscillation with a Berry phase and the Fermi level in the middle of the bandgap, the 2D WAL can be related to the topological SS.

.

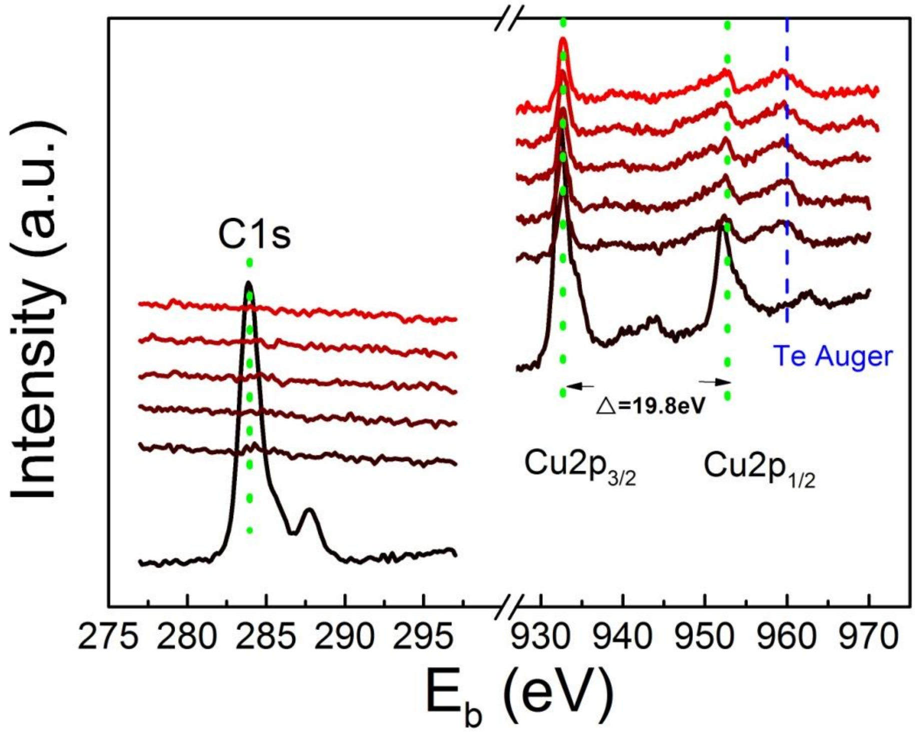

Supplemenatry Figure 3 – XPS measurement of the sample at Cu 2p state.

Here we show the XPS of the aged samples after ion milling for a few minutes, essentially exfoliated a few hundred nanometers. We can see the position of Cu 2p3/2, which confirms the zero valence of the Cu atoms in the crystal. This agrees with our model of Cu clusters.

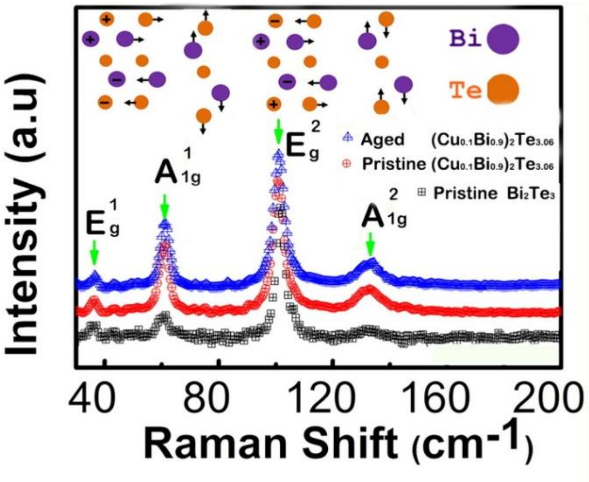

Supplementary Figure 4 – Raman measurements.

Here we show the Raman spectra for pristine Bi_2Te_3, $(Cu_{0.1}Bi_{0.9})_2Te_{3.06}$ and aged $(Cu_{0.1}Bi_{0.9})_2Te_{3.06}$ crystals. One may see four peaks, i.e. $E^1{}_g$=36.5cm^{-1}, $A^1{}_{1g}$=62.0cm^{-1}, $E^2{}_g$=102.3cm^{-1} and $A^2{}_{1g}$=134.0cm^{-1}, which appear in all of our samples with/without doping and with/without aging. Their vibration models are shown in the inset. The purple dots are bismuth atoms and the brown dots are tellurium atoms. The Raman peaks of all the samples are coincident with each other, confirming genuine crystalline structure in all our samples. This indicates that the local crystalline order is maintained in spite of the large amounts of Cu dopants and the intense aging.
